# Hepatic stellate cell-intrinsic role of SOCS1 in controlling hepatic fibrogenic response and the pro-inflammatory macrophage compartment during liver fibrosis

**DOI:** 10.3389/fimmu.2023.1259246

**Published:** 2023-10-04

**Authors:** Rajani Kandhi, Mehdi Yeganeh, Akihiko Yoshimura, Alfredo Menendez, Sheela Ramanathan, Subburaj Ilangumaran

**Affiliations:** ^1^ Department of Immunology and Cell Biology, Faculty of Medicine and Health Sciences, Université de Sherbrooke, Sherbrooke, QC, Canada; ^2^ Department of Microbiology and Immunology, Keio University School of Medicine, Tokyo, Japan; ^3^ Department of Microbiology and Infectious Diseases, Faculty of Medicine and Health Sciences, Université de Sherbrooke, Sherbrooke, QC, Canada

**Keywords:** alanine transferase (ALT), carbon tetrachloride (CCl_4_), extracellular matrix (ECM), liver fibrosis (LF), matrix metalloproteinase (MMP), nanozoomer Digital Pathology (NDP), suppressor of cytokine signaling (SOCS)

## Abstract

**Introduction:**

Hepatic stellate cells (HSC) become activated, differentiate to myofibroblasts and produce extracellular fibrillar matrix during liver fibrosis. The hepatic fibrogenic response is orchestrated by reciprocal interactions between HSCs and macrophages and their secreted products. SOCS1 can regulate several cytokines and growth factors implicated in liver fibrosis. Here we investigated the role of SOCS1 in regulating HSC activation.

**Methods:**

Mice lacking SOCS1 in HSCs (*Socs1^ΔHSC^*) were generated by crossing *Socs1^fl/fl^* and LratCre mice. Liver fibrosis was induced by carbon tetrachloride and evaluated by Sirius red staining, hydroxyproline content and immunostaining of myofibroblasts. Gene expression of pro-fibrogenic factors, cytokines, growth factors and chemokines were quantified by RT-qPCR. The phenotype and the numbers of intrahepatic leukocyte subsets were studied by flow cytometry. The impact of fibrosis on the development of diethyl nitrosamine-induced hepatocellular carcinoma was evaluated.

**Results:**

*Socs1^ΔHSC^* mice developed more severe liver fibrosis than control Socs1fl/fl mice that was characterized by increased collagen deposition and myofibroblast differentiation. *Socs1^ΔHSC^* mice showed a significant increase in the expression of smooth muscle actin, collagens, matrix metalloproteases, cytokines, growth factors and chemokines in the liver following fibrosis induction. The fibrotic livers of *Socs1^ΔHSC^* mice displayed heightened inflammatory cell infiltration with increased proportion and numbers of Ly6ChiCCR2+ pro-inflammatory macrophages. This macrophage population contained elevated numbers of CCR2+CX3CR1+ cells, suggesting impaired transition towards restorative macrophages. Fibrosis induction following exposure to diethyl nitrosamine resulted in more numerous and larger liver tumor nodules in *Socs1^ΔHSC^* mice than in *Socs1^fl/fl^* mice.

**Discussion:**

Our findings indicate that (i) SOCS1 expression in HSCs is a critical to control liver fibrosis and development of hepatocaellular carcinoma, and (ii) attenuation of HSC activation by SOCS1 regulates pro-inflammatory macrophage recruitment and differentiation during liver fibrosis.

## Introduction

Liver fibrosis is a leading cause of global morbidity and mortality (1). Liver fibrosis also promotes the development of hepatocellular carcinoma (HCC), a major cause of cancer related mortality (2, 3). Hepatocyte damage caused by viral pathogens, toxic chemicals and metabolites and metabolic overload induce an inflammatory response in the liver to contain and repair the damage (4–6). Mediators released by stressed and dying hepatocytes and cellular debris activate hepatic stellate cells (HSC) and Kupffer cells to initiate an inflammatory response. Monocyte-derived macrophages and other recruited immune cells dynamically participate in liver inflammation and tissue repair (7, 8). The tissue repair process requires production of extracellular matrix (ECM) components such as collagens by myofibroblasts, which arise mainly from HSCs, to aid hepatocyte proliferation and tissue regeneration. However, the hepatic fibrogenic response can become exaggerated with persistent hepatocyte damage, resulting in chronic inflammation, myofibroblast proliferation, excess ECM production and replacement of the liver parenchyma with fibrous connective tissue. Even though liver fibrosis is reversible at early stages, progressive liver fibrosis can lead to liver cirrhosis and loss of vital hepatic functions. HCC predominantly arises in cirrhotic livers, as compensatory hepatocyte proliferation within the prevailing inflammatory milieu facilitates acquisition of genetic lesions and neoplastic transformation (9, 10). Given the clinical significance of liver fibrosis and HCC, intense efforts are being made to halt the progression of liver fibrosis towards cirrhosis and HCC through greater understanding of the cellular and molecular mediators and the underlying mechanisms (11–14). Various animal models of experimental liver fibrosis and HCC induction have made immense contributions to this endeavor (15–17).

HSCs are central players in liver fibrosis development and progression, first as a sensor of mediators released by damaged hepatocytes and activated macrophages, and second as the main source of matrix-producing myofibroblasts (14). HSCs are situated in the space of Disse in close contact with hepatocytes on one side and endothelial cells and the associated Kupffer cells on the sinusoidal side (18). In normal livers, HSCs are maintained in a non-proliferative quiescent state and function as the principal cell type for storing retinyl esters as cytoplasmic lipid droplets. Upon activation, HSCs lose these vitamin A droplets and transdifferentiate to contractile myofibroblasts that proliferate, express fibrogenic genes and deposit ECM proteins. The activation state of HSCs is perpetuated by several chemokines, cytokines, growth factors and other soluble mediators produced by stressed hepatocytes, biliary epithelial cells, Kupffer cells, recruited proinflammatory macrophages, liver sinusoidal endothelial cells and platelets, whereas IFNγ produced by natural killer (NK) and NKT cells inhibits HSC activation (14, 19). Upon cessation of the inflammatory stimuli, pro-resolution macrophages play a crucial role in reversing the fibrotic changes by inhibiting HSC activation and by producing enzymes that clear the fibrillar matrix. Fibrosis resolution also involves clearance of activated HSCs through cellular senescence and apoptosis. Targeting the cytokines, chemokines and growth factors that promote HSC activation is a promising strategy for therapeutic intervention of liver fibrosis (12, 20).

Cytokines and growth factors that activate HSCs are mainly regulated by mechanisms that control their signaling (21). Suppressor of cytokine signaling 1 (SOCS1) is an indispensable regulator of IFNγ signaling and can also inhibit signaling by many other cytokines and growth factors (22, 23). SOCS1 is also critical to control the production of inflammatory cytokines and chemokines such as IL-6, TNFα and MCP-1/CCL2 by activated macrophages (24). Low SOCS1 expression correlates with increased fibrosis in human patients with chronic liver disease, and *Socs1* haplo-insufficient mice display increased susceptibility to fibrosis induction by dimethynitrosamine (25). We have shown that whole body SOCS1-deficient mice in an IFNγ-deficient background are highly susceptible to liver fibrosis induction following carbon tetrachloride (CCl_4_)-induced necro-inflammatory chemical injury (26). We have also shown that selective ablation of SOCS1 in myeloid cells results in heightened sensitivity to liver fibrosis, indicating a key role of SOCS1 in regulating cytokine responses in macrophages during hepatic fibrogenic response (27). As macrophages release many cytokines and growth factors that directly impact HSCs during liver fibrosis (8, 14), we ablated the *Socs1* gene selectively in HSCs using the Cre recombinase expressed under the promoter of lecithin retinol acyltransferase (LRAT) involved in retinol storage in HSCs (28, 29). In the current study, we show that HSC-specific SOCS1 deletion worsens liver fibrosis via enhancing HSC activation and by promoting the accumulation of proinflammatory macrophages, which suggest that SOCS1 expression in HSCs is crucial to control the reciprocal crosstalk between activated HSCs and macrophages.

## Materials and methods

### Mouse strains

*Socs1^fl/fl^* mice (30) were backcrossed to C57BL/6 mice obtained from Charles River Laboratories for more than ten generations. *Lrat^Cre^* mice were obtained from Dr. C. Österreicher (University of Vienna) (29) and rederived by cesarian section into the specific pathogen-free facility in our animal colony. *R26^ZsGreen^* reporter mice (Jax mice: 007906; B6. Cg-Gt(ROSA)26Sor tm6(CAG-ZsGreen1) Hze/J; also known as Ai6 mouse) (31) were obtained from the Jackson Laboratory. The *R26^ZsGreen^* reporter mouse harbors a targeted mutation at the Rosa 26 locus (Gt (ROSA)26S) with a loxP-flanked STOP cassette, which prevents the transcription of a CAG promoter-driven enhanced green fluorescent protein ZsGreen1. *Lrat^Cre^* mice were bred with the *R26^ZsGreen^* reporter to verify *Lrat^Cre^*-induced ZsGreen expression in HSCs. *Socs1^fl/fl^* mice were bred with *Lrat^Cre^* mice to generate *Socs1^fl/fl^Lrat^Cre^* mice lacking SOCS1 expression in HSCs (*Socs1^ΔHSC^*). Mice were housed in ventilated cages with 12 hours day/night cycle and fed with normal chow *ad libitum*. All experiments on mice were carried out during daytime with the approval of the Université de Sherbrooke Ethics Committee for Animal Care and Use (Protocol ID: 2018-2083).

### Induction of liver fibrosis

To induce liver fibrosis, carbon tetrachloride (CCl_4_; Sigma-Aldrich, Oakville, ON) diluted at 1:3 ratio in corn oil was administered via intra-peritoneal route (0.5 μL/g body weight) in 8-10-week-old mice twice a week for five weeks (32). Only male mice were used in this study due to the protective effect of estrogens on inflammatory cytokine production in female mice (33–35). Three days after the last injection, mice were euthanized, and serum and liver tissues were collected. Liver tissues were processed for histology, protein and mRNA expression and flow cytometry analyses of intrahepatic leukocytes, as detailed below.

### Measurement of serum ALT and liver hydroxyproline content

Serum alanine transferase (ALT) levels were measured using a kinetic assay kit from Pointe Scientific Inc. (Brussels, Belgium) following manufacturer’s instructions. Hydroxyproline content in liver tissue homogenates was measured as described previously (26).

### Histology

Pieces of liver tissues were fixed in 4% paraformaldehyde overnight and stored in 70% ethanol until they were processed for paraffin embedding following standard methods. Sections of formalin-fixed paraffin embedded liver tissues (5 µm) were deparaffinized, rehydrated, and stained with hematoxylin and eosin (H&E) or Sirius red as previously described (26). Digital images of stained sections were acquired using a Nanozoomer Digital Pathology (NDP) slide scanner and analyzed using the NDP.view2 software (Hamamatsu Photonics, Japan). Quantification of Sirius red staining areas was done using the Image J software (National Institutes of Health, Bethesda, MD, USA) from twenty randomly selected fields from three to five mice in each group.

### Immunohistochemistry and immunofluorescence

Immunohistochemical (IHC) staining of liver sections for alpha smooth muscle actin (αSMA) was done as previously described to detect myofibroblasts (26) (**Supplementary Table S1**). Macrophages were detected by immunofluorescence (IF) staining. Deparaffinized tissue sections were incubated overnight with CD68 antibody (**Supplementary Table S1**) followed by a AlexaFluor-488-conjugated secondary antibody (Invitrogen/ThermoFisher Scientific) for 1 h at room temperature in the dark. Nuclei were stained with Hoechst 33342 (Thermo Fisher; Cat# 62249) for 5 min at room temperature. The stained slides were washed and mounted in Vectashield (Vector Laboratories; Cat# H-1900) antifading medium. IHC images were captured using the NDP slide scanner and IF images acquired using Axioskop 2 fluorescence microscope (Carl Zeiss Canada Ltd, Toronto, Canada). Staining intensity of αSMA and the proportion of CD68 positive cells were quantified in three randomly selected fields from three to five mice in each group using the NIH ImageJ software.

### Gene expression analysis

RNA extraction, cDNA preparation and gene expression analysis by RT-qPCR were carried out as described previously (26). All RT-qPCR primers (**Supplementary Table S2**) showed more than 90% efficiency and displayed a single melting curve. Expression levels of specific genes were normalized for the housekeeping gene *Rplp0* (36B4) within each experimental group and expressed as fold induction compared to the control group.

### Western blot

Liver tissue lystaes were prepared from snap frozen samples using a tissue homogenizer bead mill (MM 400; Retsch, Hann, Germany) and protein concentration determined as previously described (26). Thirty μg of total protein from each sample were separated on SDS-polyacrylamide gels, blotted on to PVDF membrane and probed for the indicated proteins using the primary antibodies listed in **Supplementary Table S3**. HRP-conjugated mouse and rabbit secondary antibodies and enhanced chemiluminescence reagents (GE Healthcare Life Sciences, Pittsburg, PA) were used to reveal the western blot bands. Images were captured using the VersaDOC 5000 imaging system (Bio-Rad).

### Primary HSC isolation, culture and activation

HSCs were isolated from 12-weeks old mice by equilibrium density gradient centrifugation of liver cells released by collagenase digestion, following published methods with some modifications (36, 37). Mice were anesthetized by intraperitoneal administration of Ketamine-Xylazine mixture (Ketamine-87 mg/kg; Xylazine-13 mg/kg - in normal saline), placed on supine position, the liver was exposed and the inferior vena cava canulated as described by Mederacke et al. (37). The livers were perfused with 0.5 mM EGTA in HEPES-buffered Hank’s balanced salt solution (HHBSS: NaCl 140 mM, KCl 5.4 mM, Na_2_HPO_4_ 0.34 mM, NaHCO_3_ 4.2 mM, KH_2_PO_4_ 0.44 mM; MgCl_2_ 0.4 mM, HEPES 10 mM, D-glucose 100 mg/L) without calcium, maintained at 42°C in a water bath, for 2 min using a peristaltic pump, with the portal vein severed to flush out erythrocytes. The liver parenchyma was digested by perfusion with freshly prepared Type IV collagenase (Worthington Biochemical; Cat # LS004186; 18 mg of 325 collagen digestion units) in 50 mL of prewarmed HHBSS containing 1.5 mM CaCl_2_ (wash buffer). Tissue digestion was stopped when the liver became amorphous and collapsed (~7 min). The digested liver was carefully removed and aseptically transferred to a Petri dish. The liver tissue was teased apart using forceps to release cells into suspension, which was passed through 70 μm nylon filter strainer (BD Falcon) to remove tissue debris. Hepatocytes were sedimented by centrifuging the cell suspension at 50 g for 5 min at 4°C. Nonparenchymal cells were pelleted down by centrifuging the supernatant at 500 g for 10 min at 4°C. The cell pellet was resuspended in 5 mL 20% OptiPrep™ (Axis Shield; 60% stock diluted with wash buffer), transferred to a 15 mL tube, slowly overlaid with 5 mL 11.5% OptiPrep and then 2 mL wash buffer, and centrifuged at 1500 g for 17 min at 4°C without brake. An opaque layer formed at the interface between 11.5% OptiPrep and the wash buffer was carefully collected with a Pasteur pipette and the cells were washed at 500 g for 5 min at 4°C. The cells were resuspended in DMEM containing 10% FCS and counted.

For IF microscopy, HSCs were seeded on coverslips in a 12 well plate at 1 ×10^5^ cells/well in DMEM-10% FCS and cultured with the indicated cytokines and growth factors. Control and stimulated cells on coverslips were washed in phosphate-buffered saline (PBS), fixed in ice-cold methanol for 5 min at room temperature, followed by washing in PBS three times each for 5 min. The coverslips were incubated in PBST (PBS with 0.2% Triton X-100) containing 5% BSA for 1 h to block non-specific Ab binding. This was followed by incubation with primary Ab diluted in PBST-1% BSA in a humidified chamber for 1 h at room temperature or overnight at 4°C. Subsequent steps were similar to those described for IF microscopy of tissue sections.

For gene expression studies, control HSCs were lysed immediately after isolation in RNAlater (ThermoFisher Scientific). For stimulation with cytokines and growth factors, primary HSCs were cultured in 35 mm Petri dishes (0.5 ×10^6^ cells/well) and cultured in DMEM-10% FCS in the presence of IL-6 (10 ng/mL), TGFβ (5 ng/mL) or PDGFB (20 ng/mL) (all from R&D Sytems, Minniapolis, MN). After for 24 h incubation, the cells were lysed in RNAlater for gene expression analysis as previously described (26).

### Isolation of intrahepatic leukocytes and flow cytometry

IHLs were isolated by a four steps protocol involving (i) controlled collagenase digestion of minced liver tissue using the GentleMACS tissue dissociator device (Miltenyi Biotech), (ii) microfiltration and sedimentation of hepatocytes and IHLs by differential centrifugation, (iii) Percoll gradient centrifugation of sedimented IHLs to remove fatty debris, and (iv) magnetic selection of hematopoietic cells using anti-CD45 antibody to clarify the resuspended Percoll-sedimented cells as detailed elsewhere (38). This IHL isolation procedure developed for fatty liver tissues is applicable to fibrotic and normal livers. The cells were resuspended in PBS containing 2% fetal bovine serum (FBS) for flow cytometry analysis.

Aliquots of IHLs were incubated in 100 μL of Fc Block diluted in PBS-2%FBS for 10 min on ice. After washing in PBS-2%FBS, the cells are incubated with a panel of fluorochrome conjugated antibodies (**Supplementary Table S4**) diluted in PBS-2%FBS. The cells were washed and fluorescence data were acquired using the CytoFlex flow cytometer (Beckman Coulter). The data was analyzed using the FlowJo software (BD Biosciences).

### Induction of HCC

To induce HCC, 2 weeks old male mice were injected via intra peritoneal route diethylnitrosamine (DEN; Millipore-Sigma; 25 mg/Kg body weight) followed by bi-weekly injections of CCl_4_ (0.5 ml/Kg) starting at 8 weeks of age for 14 consecutive weeks (39). Mice were euthanized after 22 weeks and tumor development assessed by counting the number of nodules and measuring the liver/body weight ratio.

### Statistical analyses

The data were compiled on an Excel spreadsheet (Microsoft 365) and Prism V9.3.1 software (GraphPad, La Jolla, CA, USA) was used to plot graphs and for statistical analysis. *p* values <0.05 were considered significant.

## Results

### SOCS1 loss in HSCs exacerbates chemically induced liver fibrosis

Cytokines and growth factors produced by stressed hepatocytes and activated macrophages are the key drivers of liver fibrosis and represent potential therapeutic targets (4, 12). The loss of SOCS1, a key regulator of inflammatory cytokine and growth factor signaling in the liver, promotes liver fibrosis (25, 26, 40). Primary HSCs isolated from SOCS1-deficient mice display increased proliferation in response to IL-6, PDGF, EGF, TGFα and HGF, suggesting a cell-intrinsic role of SOCS1 in regulating HSC activation (26). To directly assess the role of SOCS1 in regulating HSCs responses during liver fibrosis, we crossed *Socs1^fl/fl^* mice with *Lrat^Cre^* deleter mice, which ablates floxed genes specifically in HSCs in the liver (28, 29). We confirmed the expression of *Lrat* promoter driven Cre expression in HSCs using the ROSA26-ZsGreen reporter mice (31). Cryosections of livers from *Lrat^Cre^* ROSA26-ZsGreen mice showed a ZsGreen expression pattern that is consistent with the distribution of HSCs (**Supplementary Figure S1A**), which was confirmed by immunostaining for the HSC marker desmin (**Supplementary Figure S1B**) as well as by verifying ZsGreen expression in primary HSCs (**Supplementary Figure S1C-E**).

**Figure 1:**
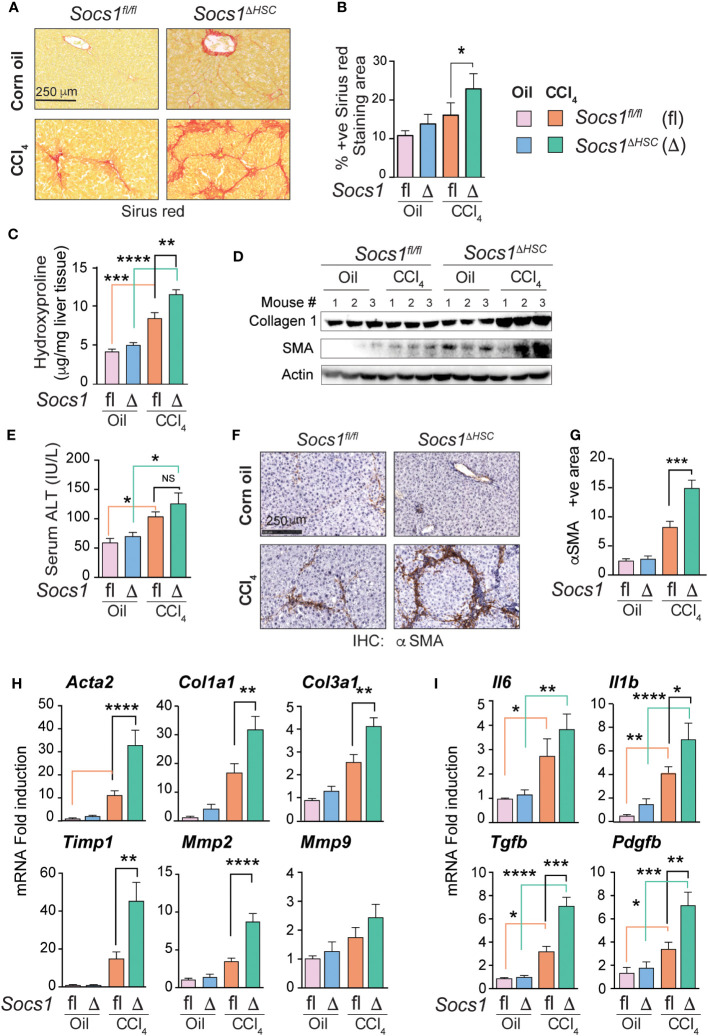
SOCS1 deficiency in HSCs increases the severity of liver fibrosis induced by CCl_4_. Eight weeks old male *Socs1^ΔHSC^* and sex matched *Socs1^fl/fl^* littermate controls were treated with CCl_4_ or vehicle (corn oil) twice a week for five weeks and euthanized three days later. **(A)** Sirius red staining for collagen deposition. Representative data from at least three mice for each group are shown. **(B)** Quantification of sirius red staining areas. Mean ± standard error of mean (SE) from three to six mice per group for two independent experiments. **(C)** Hydroxy proline content of liver tissues. Mean ± SE from five to eight mice per group from two experiments. **(D)** Western blot evaluation of collagen 1 and αSMA proteins in the liver tissues from three mice in each group. Beta actin was used as a loading control. **(E)** Serum ALT levels from four mice per group from two different experiments are shown (mean ± SE). **(F)** IHC staining of αSMA in representative liver sections. Representative data from at least three mice in each group are shown. **(G)** Quantification of αSMA staining area. Mean ± SE from three to five mice per group from two experiments. **(H, I)** RT-qPCR evaluation of the expression of genes associated with hepatic fibrogenic response **(H)**, cytokines and growth factors **(I)**. n = 6-8 mice per group from a minimum of two separate experiments. Statistical significance was assessed by one-way ANOVA with Tukey’s multiple comparison test. ns, not significant; * *p <*0.05, ** *p <*0.01, *** *p <*0.001; **** *p <*0.0001.

Liver fibrosis was induced in *Socs1^ΔHSC^ and Socs1^fl/fl^* control mice by intraperitoneal administration of CCl_4_ twice a week for 5 weeks. Sirius red staining of the liver sections of these mice showed increased collagen deposition with prominent bridging fibrosis pattern compared to limited septal fibrosis observed in *Socs1^fl/fl^* control mice (**Figure 1A**). Quantification of the staining area and intensity showed significantly elevated levels of collagen deposition in HSC-specific SOCS1-deficient mice livers that was confirmed by hepatic hydroxyproline content (**Figure 1B, C**), Masson’s trichrome staining and western blot (**Supplementary Figure S2** and **Figure 1D**). However, the increase in serum ALT levels following CCl_4_ treatment was comparable between *Socs1^ΔHSC^* and *Socs1^fl/fl^* control mice (**Figure 1E**), suggesting that increased fibrosis in *Socs1^ΔHSC^* mice resulted from increased fibrogenic response caused by the loss of SOCS1 in HSCs rather than from increased liver damage. This was confirmed by immunohistochemical staining of αSMA in myofibroblasts, which showed intense staining and significantly increased staining area in *Socs1^ΔHSC^* mice compared to *Socs1^fl/fl^* controls (**Figure 1F, G**). Consistent with this data, CCl_4_-treated *Socs1^ΔHSC^* mice livers showed increased expression of *Acta2* and *Col1a1* genes and αSMA and collagen 1 protein expression (**Figure 1H, D**). The fibrotic livers of *Socs1^ΔHSC^* mice showed significantly elevated expression of *Col3a1*, the antifibrotic matrix metalloproteinase 2 (*Mmp2*) (41) and tissue inhibitor of MMPs 1 (*Timp1*) genes compared to *Socs1^fl/fl^* mice livers (**Figure 1H**). Moreover, expression of genes coding for proinflammatory cytokines IL-6 and IL-1β, profibrogenic TGFβ and the myofibroblast growth factor PDGFB, which were markedly induced in *Socs1^fl/fl^* mice livers, was significantly elevated in the fibrotic livers of *Socs1^ΔHSC^* mice (**Figure 1I**). These data indicated that SOCS1 expression in HSCs plays a crucial role in regulating hepatic fibrogenic response induced by chemical agents.

### SOCS1-deficient HSCs display increased responsiveness to TGFβ stimulation

**Figure 2:**
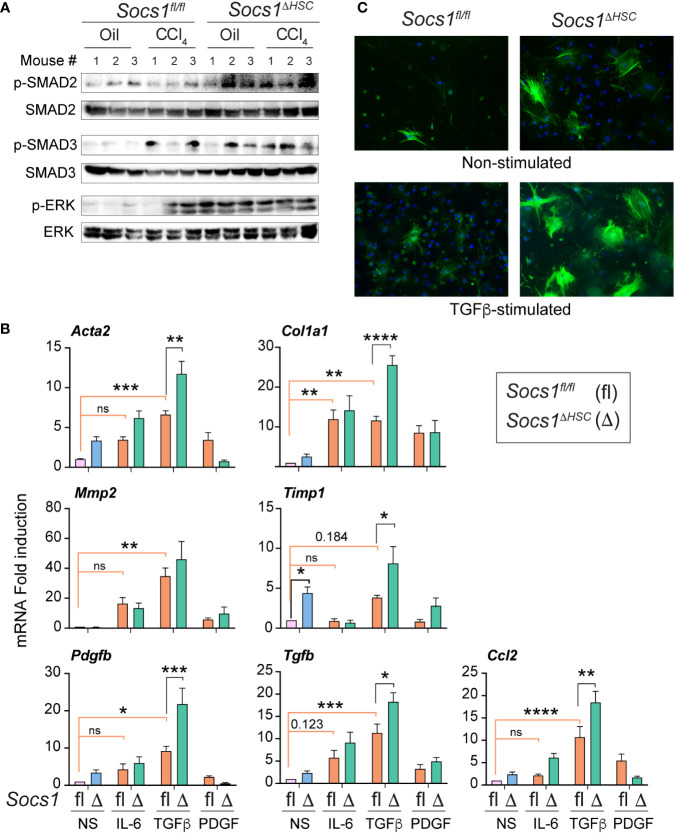
Primary HSCs from *Socs1^ΔHSC^* mice show increased responsiveness to TGFβ stimulation. **(A)** Phosphorylated and total SMAD2, SMAD3 and ERK1/2 protein levels in the liver tissues from three mice in the indicated groups. **(B)** RT-qPCR evaluation of the expression of genes associated with hepatic fibrogenic response following stimulation with IL-6 (10 ng/mL), TGFβ (5 ng/mL) or PDGFB (20 ng/mL) for 24 h. Mean ± SE of data was pooled from in triplicate cultures from two experiments were compared by one-way ANOVA with Tukey’s multiple comparison test. * *p <*0.05, ** *p <*0.01, *** *p <*0.001; **** *p <*0.0001. ns, not significant. **(C)** IF evaluation of αSMA expression in four days old primary HSC cultures from *Socs1^fl/fl^* and *Socs1^ΔHSC^* mice without or with TGFβ stimulation (5 ng/mL) for 24 h. Representative images from two experiments are shown.

As TGFβ is a key driver of ECM deposition (42, 43), we examined TGFβ signaling pathway components in the fibrotic livers of *Socs1^ΔHSC^ and Socs1^fl/fl^* mice. *Socs1^ΔHSC^* mice displayed increased phosphorylation of SMAD2 and SMAD3 in both CCl_4_ and oil treated groups (**Figure 2A**). On the other hand, increased SMAD3 phosphorylation was observed in the livers of *Socs1^fl/fl^* mice only after CCl_4_ treatment, whereas SMAD2 phosphorylation level was not altered (**Figure 2A**). Similarly, phosphorylation of the MAP kinase ERK1/2 was prominent in both in both CCl_4_ and oil treated groups of *Socs1^ΔHSC^* mice but occurred only after CCl_4_ treatment in control mice (**Figure 2A**). These observations suggested increased responsiveness of SOCS1-deficient HSCs to TGFβ and growth factor signaling that promoted their fibrogenic response. To test this possibility, primary HSCs enriched from *Socs1^ΔHSC^* and *Socs1^fl/fl^* mice were exposed to inflammatory (IL-6), fibrogenic (TGFβ) and growth stimulating (PDGFB) cytokines and the induction genes that promote myofibroblast differentiation (*Acta2*) and matrix production (*Col1a1*) and remodelling (*Mmp2*, *Timp1*) was evaluated. TGFβ strongly induced *Acta2*, *Col1a1*, *Mmp2* and *Timp1* genes in control HSCs and the induction of all but *Mmp2* were further amplified significantly by SOCS1 deficiency (**Figure 2B**). Whereas IL-6 caused a discernible increase in the expression of *Acta2*, *Col1a1* and *Mmp2* genes in control and SOCS1-deficient HSCs, PDGFB, the most potent growth factor for HSCs (18, 44), did not change the expression of these fibrogenic genes. Notably, SOCS1-deficient HSCs showed discernibly elevated basal expression of *Acta2* and *Timp1* genes (**Figure 2B**), suggesting production of autocrine fibrogenic mediators in SOCS1-deficient HSCs. Indeed, TGFβ stimulation upregulated *Tgfb* and *Pdgfb* genes in control HSCs that was significantly amplified by SOCS1 deficiency (**Figure 2B**). Immunofluorescence staining of αSMA following TGFβ stimulation showed profound increase in SOCS1-deficient HSCs compared to control HSCs (**Figure 2C**). These results indicated that SOCS1 is a critical to regulator of HSC activation by limiting their responsiveness to TGFβ stimulation and TGFβ-induced autocrine mediator production.

### SOCS1 expression in HSCs regulates inflammatory cell recruitment during liver fibrosis

**Figure 3:**
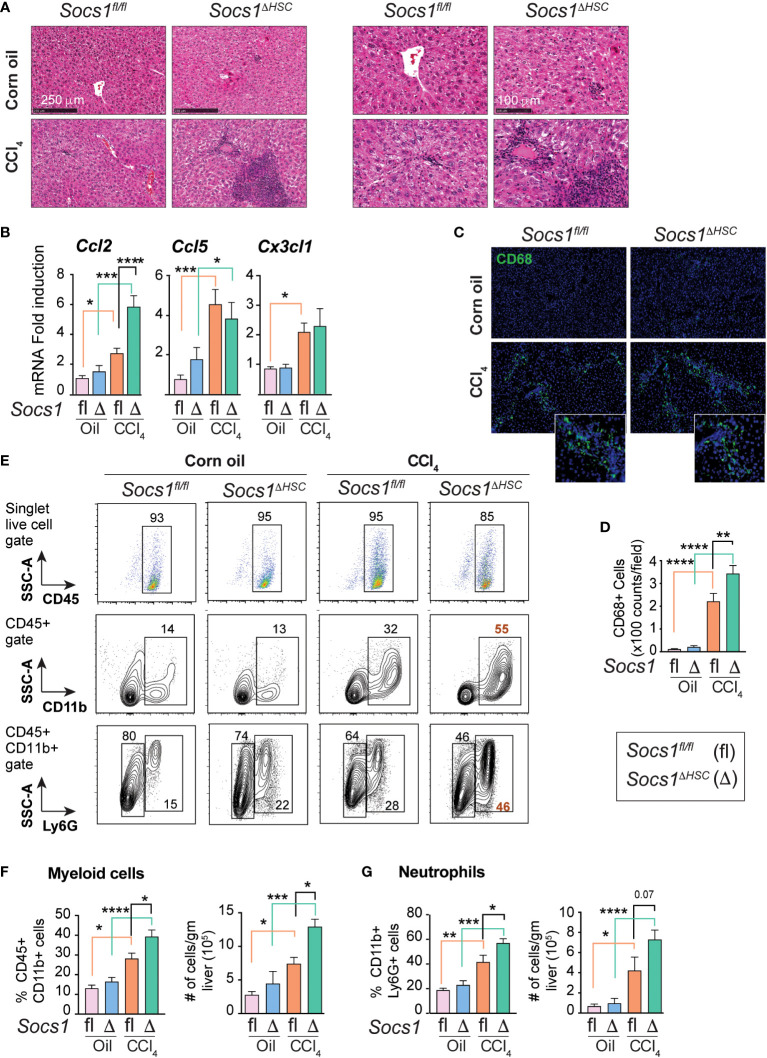
Fibrotic livers of HSC-specific SOCS1 deficient mice show increased innate immune cell infiltration. **(A)** Representative hematoxylin and eosin-stained liver sections of *Socs1^fl/fl^* and *Socs1^ΔHSC^* mice treated with corn oil or CCl_4_ at lower (left panels) and higher (right panels) magnification. **(B)** RT-qPCR evaluation of chemokine gene expression in liver tissues. Mean ± SE from 6-8 mice per group from at least two separate experiments **(C)** CD68 IF staining of representative liver sections. Insets show magnified images for CCl_4_-treated mice livers. **(D)** Quantification of CD68+ cells. Mean ± SE quantified from 8-10 fields from 3-4 mice/group. **(E)** Immunophenotyping of IHLs from corn oil or CCl_4_ treated mice groups by flow cytometry. Representative zebra blots showing distribution of CD45+, CD45+CD11b+ and CD45+CD11b+Ly6G+ cells. Numbers inside the plots indicate the proportion of cells within the gated cell populations. **(F, G)** Proportions and absolute counts of CD45+CD11b+ myeloid cells **(F)** and CD45+CD11b+Ly6G+ polymorphonuclear cells **(G)** in the indicated groups of mice. Pooled data from 5-7 mice per group from two different experiments are shown (mean ± SE). One-way ANOVA with Tukey’s multiple comparison test. * *p <*0.05, ** *p <*0.01, *** *p <*0.001; **** *p <*0.0001.

Hematoxylin and eosin-stained liver sections of CCl_4_-treated mice revealed increased inflammatory cell infiltration in *Socs1^ΔHSC^* mice in the periportal area and around the central vein compared to *Socs1^fl/fl^* controls (**Figure 3A**). This observation suggested that SOCS1 deficiency in HSCs enhances hepatic inflammatory response during fibrogenesis. In support of this notion, the expression of the *Ccl2* chemokine gene, which is strongly induced by CCL_4_ treatment in control mice, was further increased in HSC-specific SOCS1-deficient mice (**Figure 3B**). However, the induction of *Ccl5* (RANTES) and *Cx3cl1* (Fractalkine) genes was comparable between *Socs1^ΔHSC^* and *Socs1^fl/fl^* mice livers. We also observed that TGFβ strongly induced the *Ccl2* gene in primary HSCs that was significantly elevated in SOCS1-deficient HSCs (**Figure 2B**). As *Ccl2* encodes the macrophage chemoattractant protein 1 (MCP1/CCL2), we examined the distribution of macrophages in the CCL_4_-treated livers. The fibrotic livers of *Socs1^fl/fl^* mice harbored significantly more CD68+ cells, possibly representing Kupffer cells arising from recruited macrophages (45), and their numbers increased further in *Socs1^ΔHSC^* mice compared to *Socs1^fl/fl^* mice (**Figure 3C, D**). Flow cytometry analysis of intrahepatic leukocytes revealed that the proportion and number of total CD45+CD11b+ myeloid cells and CD11b+Ly6G+ polymorphonuclear neutrophils, which were comparable between vehicle-treated *Socs1^ΔHSC^* and *Socs1^fl/fl^* mice, significantly increased following fibrosis induction and this increase was further augmented by SOCS1 deficiency in HSCs (**Figure 3E–G**). These data indicated that SOCS1 expression in HSCs is crucial to control immune cell recruitment and to regulate fibrosis-associated inflammatory response during liver fibrosis.

### SOCS1 loss in HSCs promotes a pro-inflammatory macrophage phenotype

**Figure 4:**
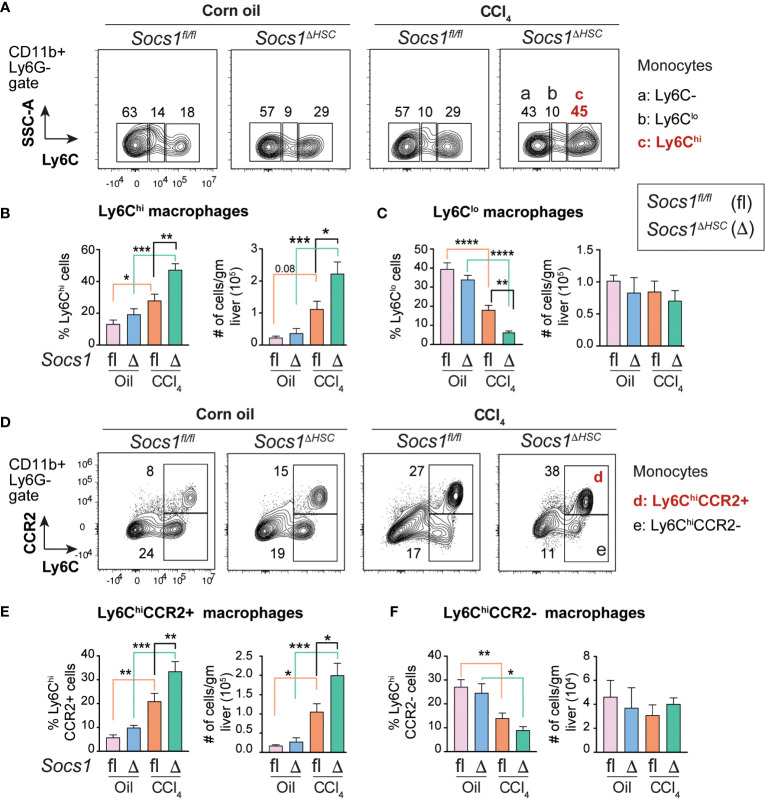
Increased inflammatory macrophage infiltration in the fibrotic livers of HSC-specific SOCS1 deficient mice. **(A)** Representative density blots showing expression of the proinflammatory monocyte marker Ly6C within the CD45^+^CD11b^+^Ly6G− cells in the livers of *Socs1^ΔHSC^* and *Socs1^fl/fl^* control mice. Gates represented by alphabets identify (a) Ly6C− (b) Ly6C^lo^ and (c) Ly6C^hi^ macrophage populations, and the numbers indicate their proportions within the gates. **(B, C)** Proportions and absolute counts of Ly6G−Ly6C^hi^ proinflammatory macrophages compared to the Ly6G−Ly6C^lo^ subset. **(D)** Representative zebra blots showing expression of the proinflammatory macrophage marker CCR2 within the CD45+CD11b+Ly6G−Ly6C^hi^ macrophage population in the livers of *Socs1^ΔHSC^* and *Socs1^fl/fl^* control mice. Gates represented by alphabets identify (d) CCR2+ and (e) CCR2− cell populations, and the numbers indicate their proportions within the gates. **(E, F)** Proportions and absolute counts of Ly6C^hi^CCR2+ and Ly6C^hi^CCR2− subsets. Mean ± SE data was pooled from 5-7 mice per group from two different experiments and compared by one-way ANOVA with Tukey’s multiple comparison test. * *p <*0.05, ** *p <*0.01, *** *p <*0.001; **** *p <*0.0001.

The increased numbers of myeloid cells (**Figure 3E, F**) and the heightened induction of *Il6*, *Il1b*, *Tgfb* and *Pdgfb* genes in CCl_4_-treated livers of *Socs1^ΔHSC^* mice (**Figure 1I**), suggested potent activation of monocyte-derived macrophages. To test this hypothesis, we evaluated the expression level of proinflammatory macrophage marker Ly6C (45) on CD45+CD11b+ cells (**Figure 4A**). The fibrotic livers of *Socs1^fl/fl^* mice livers harbored increased proportion and number of Ly6C^hi^ proinflammatory macrophages that were further increased in the livers of *Socs1^ΔHSC^* mice (**Figure 4B**). Even though Ly6C^lo^ anti-inflammatory macrophages were significantly reduced in frequency in the fibrotic livers of both *Socs1^fl/fl^* and *Socs1^ΔHSC^* mice, absolute number of this macrophage subset was not significantly altered (**Figure 4C**). Proinflammatory macrophages are also characterized by the upregulation the chemokine receptor CCR2 (45, 46). When the expression of CCR2 and Ly6C was examined on CD11b+Ly6G- cells, we observed a significant increase in the proportion and number of Ly6C^hi^CCR2+ cells in CCl_4_-treated livers of *Socs1^fl/fl^* mice that was further increased in *Socs1^ΔHSC^* mice (**Figure 4D, E**). The proportion of Ly6C^hi^ cells that did not express CCR2 significantly decreased in both *Socs1^fl/fl^* and *Socs1^ΔHSC^* mice, although their absolute numbers of were not significantly affected (**Figure 4F**).

### CCR2+CX3CR1+ macrophages accumulate in the fibrotic livers of *Socs1^ΔHSC^* mice

**Figure 5:**
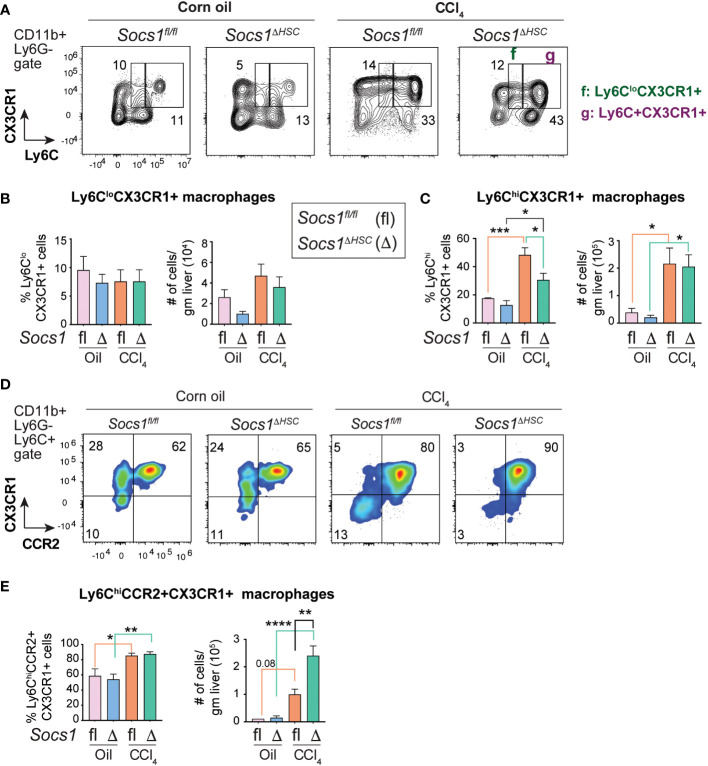
Fibrotic livers of HSC-specific SOCS1 deficient mice accumulate macrophages expressing both CCR2 and CX3CR1. **(A)** Representative zebra blots showing the expression of Ly6C and CX3CR1 within the CD45+CD11b+Ly6G− cells in the livers of *Socs1^ΔHSC^* and *Socs1^fl/fl^* control mice. Gates represented by alphabets identify (f) Ly6C^lo^CX3CR1+ and (g) Ly6C^hi^CX3CR1+ macrophage populations, and the numbers indicate their proportions within the gates. **(B, C)** Proportions and absolute counts of) Ly6C^lo^CX3CR1+ pro-resolution macrophages compared to the Ly6C+CX3CR1+ proinflammatory subset. **(D)** Representative density blots showing the expression of CCR2 and CX3CR1 within the CD45+CD11b+Ly6C+ cells in the fibrotic livers of *Socs1^ΔHSC^* and *Socs1^fl/fl^* control mice. **(E)** Proportions and absolute counts of Ly6C+CCR2+CX3CR1+ macrophages. Pooled data from 4-6 mice per group from two different experiments are shown (mean ± SEM). One-way ANOVA with Tukey’s multiple comparison test. * *p <*0.05, ** *p <*0.01, *** *p <*0.001, **** *p <*0.0001.

During fibrosis progression proinflammatory macrophages transition to restorative macrophages that promote tissue repair and fibrosis resolution after cessation of the inflammatory stimuli (45). The pro-resolution macrophages are characterized by the expression of the CX3CR1 chemokine receptor. Segregation of the macrophage population based on the expression of Ly6C and CX3CR1 revealed that the proportion of Ly6C^lo^CX3CR1+ pro-resolution macrophages did not change in the livers of CCl_4_-treated *Socs1^fl/fl^* and *Socs1^ΔHSC^* mice although their numbers showed a discernible, though not significant, increase in both groups (**Figure 5A, B** **)**. On the other hand, the proportion and number of Ly6C^hi^ cells that also expressed CX3CR1 was markedly upregulated in the fibrotic livers of *Socs1^fl/fl^* and *Socs1^ΔHSC^* mice, with a significant increase in both frequency and number (**Figure 5A, C** **)**. Next, we analyzed the co-expression of CCR2 and CX3CR1 within CD11b+Ly6G−Ly6C^hi^ pro-inflammatory macrophage population. We observed a marked increase in the frequency of Ly6C^hi^CCR2+CX3CR1+ cells in both *Socs1^fl/fl^* and *Socs1^ΔHSC^* mice with significantly elevated number of these cells in *Socs1^ΔHSC^* mice (**Figure 5D, E** **)**. These results suggest that the increased inflammatory response in *Socs1^ΔHSC^* mice prevents these intermediate or transitional macrophages from acquiring the CX3CR1+Ly6C^lo^CCR2− restorative macrophage phenotype.

### SOCS1 deficiency in HSCs promotes enrichment of CD11b+CD11c+ myeloid DCs and CD8+ T cells during liver fibrosis

The fibrotic livers of *Socs1^ΔHSC^* mice also contained an increased frequency and number of CD11b+CD11c+ myeloid dendritic cells (DC), whereas the number of plasmacytoid DCs were not affected (**Supplementary Figure S3**). The livers of CCl_4_-treated *Socs1^ΔHSC^* mice also harbored an elevated number of CD8+ T lymphocytes that displayed an activated effector (CD69+), effector memory (CD44^hi^CD62L^lo^) and central memory (CD44^hi^CD62L^hi^) phenotype, whereas the numbers of CD4 T and NK cells and the activation status of CD4+ T cells were not affected (**Supplementary Figures S4, S5**).

### Increased liver fibrosis promotes HCC development in *Socs1^ΔHSC^* mice

**Figure 6:**
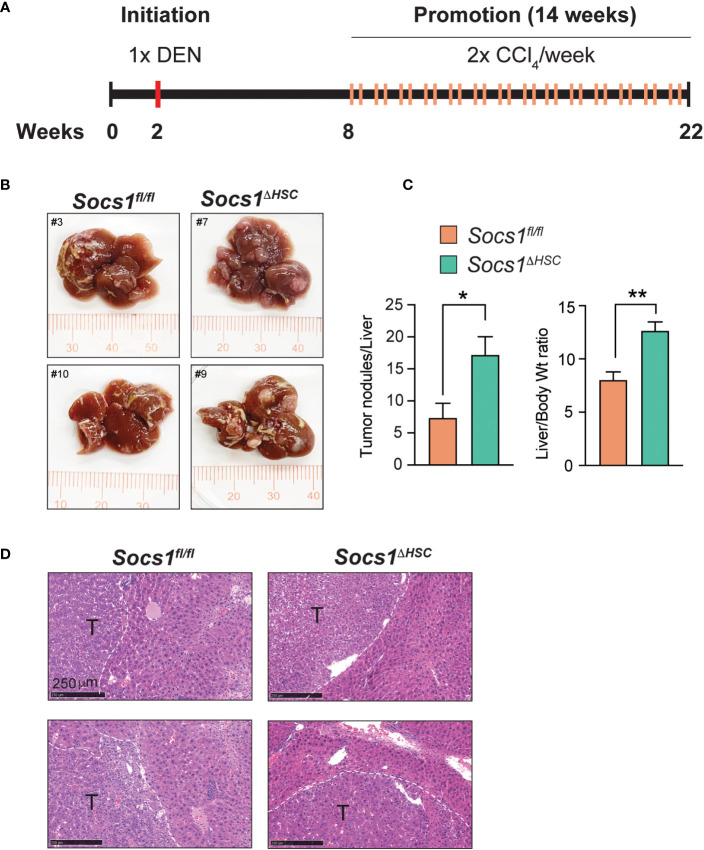
SOCS1 deficiency in HSCs enhances hepatocarcinogenesis in fibrotic livers. **(A)** Schematic representation of hepatocellular carcinoma induction. Two weeks old *Socs1^fl/fl^Lrat^Cre^* mice (n=8) and *Socs1^fl/fl^* controls (n=7) were injected with DEN (25 mg/kg BW, i.p.). Six weeks later, the mice were administered CCl_4_ (0.5 ml/kg BW, i.p.), weekly for 14 weeks, and euthanized at the age of 22 weeks. **(B)** Representative images of livers showing HCC nodules. **(C)** Liver to body weight ratio and the number of surface liver nodules per mouse. Data shown as Mean ± SE from 6-7 mice per group were compared by two-tailed unpaired t-test. * *p* < 0.05, ** *p* < 0.01. **(D)** Representative H&E sections of the tumor bearing livers. T, tumor nodule.

Liver fibrosis driven by activated HSC and the associated inflammation driven by macrophages are important drivers of HCC development and progression (10, 47, 48). To determine if increased liver fibrosis in *Socs1^ΔHSC^* mice promotes HCC development, we administered DEN to 2 weeks old mice followed by CCl_4_ treatment beginning at 8 weeks of age and continued for 14 weeks to induce and sustain liver fibrosis (**Figure 6A**). Examination of the livers at the end of this treatment period revealed increased liver body weight ratio in *Socs1^ΔHSC^* mice than in control mice, with increased number of liver tumor nodules that showed histological features of hepatocellular carcinoma (**Figure 6B–D**).

## Discussion

**Figure 7:**
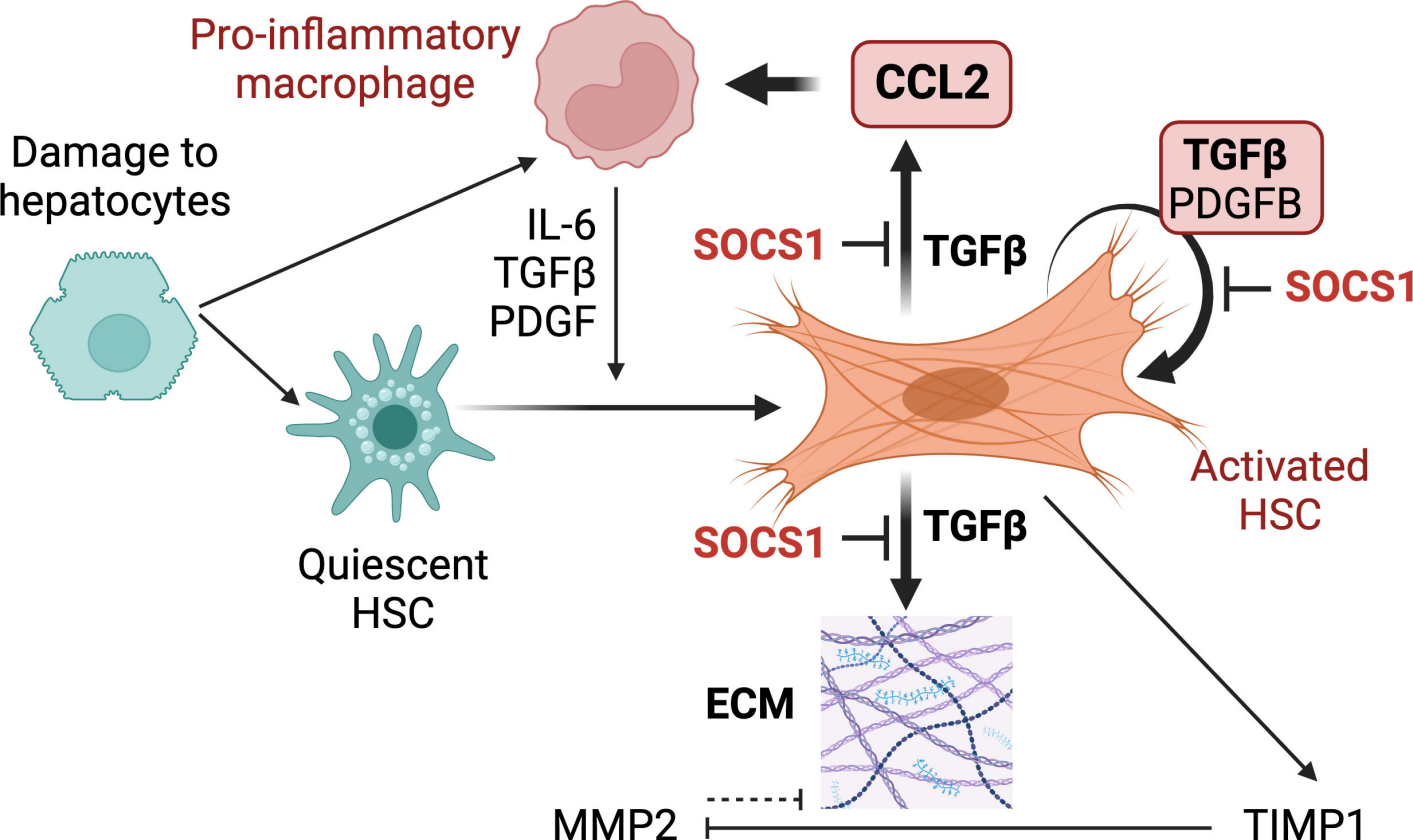
Proposed functions of SOCS1 in HSCs. Following hepatocyte damage, quiescent HSCs become activated and differentiate towards myofibroblasts to repair tissue damage. Liver resident and recruit macrophages contribute to HSC activation via secreting TGFβ, PDGF, chemokines and inflammatory cytokines. Activated HSCs produce autocrine TGFβ, which induces fibrogenic genes that promote synthesis and accumulation of ECM. TGFβ also induces autocrine PDGFB that promotes myofibroblast proliferation and induces CCL2 gene expression in HSCs that recruits macrophages and other inflammatory cells. During liver fibrosis, SOCS1 plays a crucial cell-intrinsic regulatory role in HSCs to control TGFβ-induced expression of fibrogenic and chemokine genes and autocrine TGFβ and PDGFB production.

Myofibroblasts that differentiate from HSCs are key pathogenic mediators of hepatic fibrosis that can progress towards HCC and thus are considered a key therapeutic target (10, 20, 47). Genetic targeting of HSCs has been widely used to gain deeper understanding of the molecular basis of HSC activation, differentiation and fibrogenic functions. These efforts to track HSCs or modulate their gene expression have employed mice expressing the Cre recombinase under diverse promoters such as collagens, glial fibrillary acidic protein (*Gfap*), *Pdgfb* and *Lrat* (28, 49–53). Among these, LRAT is expressed selectively in HSCs and not in hepatocytes, cholangiocytes and endothelial cells in the liver. Thus, the *Lrat^Cre^* deleter mouse has provided a valuable tool for deeper understanding of the physiopathology of liver fibrosis and HCC development (28, 29, 48, 54–58). In the current study, we ablated the *Socs1* gene using the *Lrat^Cre^* deleter to understand the role of SOCS1 in regulating HSC activation. Our findings reveal a non-redundant cell-intrinsic role of SOCS1 in HSCs that controls HSC activation by TGFβ and amplification of the hepatic inflammatory response in liver fibrosis (**Figure 7**).

SOCS1 expression can attenuate several cytokines that signal via the canonical JAK-STAT pathway as well as many growth factors that signal via receptor tyrosine kinases (22, 23, 59). Our finding that *Socs1^ΔHSC^* mice develop severe fibrosis compared to control mice indicates that SOCS1 regulates cytokines and growth factors that promote HSC activation. PDGFB, which signals via the PDGFRα and PDGFRβ receptor tyrosine kinases and activates ERK and AKT signaling pathways, is a potent paracrine and autocrine mitogen for HSCs (60). Whereas transgenic expression of PDGFB in the liver under albumin promoter increased collagen deposition and promoted CCl_4_-induced liver fibrosis, ablation of PDGFRβ in HSCs attenuated liver fibrosis (44, 61). SOCS1 was reported to interact with PDGFR (62). PDGF stimulation of primary HSCs from *Socs1^ΔHSC^* mice did not alter the expression of the fibrogenic genes (**Figure 2B**). However, we have shown that primary HSCs from whole body SOCS1 deficient mice display increased proliferation to PDGF stimulation (26), suggesting a role for SOCS1 in regulating HSC proliferation in liver fibrosis. In support of this notion, we observed increased induction of ERK1/2 phosphorylation in the livers of *Socs1^ΔHSC^* (**Figure 2A**), suggesting deregulated PDGFR signaling in SOCS1-deficient HSCs. However, further studies using *Socs1^ΔHSC^* primary HSCs are needed to determine potential contribution of other growth factors such as EGF, as it is also mitogenic to HSCs and SOCS1 can attenuate EGFR signaling (63–65).

Among the cytokines that signal via the JAK-STAT pathway, IL-6 could be involved in liver fibrosis via amplifying the intercellular communication between stressed hepatocytes, HSCs and macrophages (12). IL-6 was also reported to stimulate the expression of *Acta2* and *Col1a* expression in human HSCs (66). As SOCS1 was initially discovered as a negative regulator of the inflammatory cytokine IL-6 signaling (67), loss of SOCS1 could amplify IL-6 signaling and promote liver fibrosis. Earlier studies using whole body *Il6* knockout mice reported an anti-fibrotic role of IL-6 although another study has reported potential pro-fibrotic effects (68–70). Given that IL-6 is critical for hepatocyte survival and liver regeneration, it was postulated that decreased hepatocyte survival in the absence of IL-6 resulted in sustained hepatocyte damage, contributing to increased fibrosis in Il6-deficient mice rather than a direct anti-fibrotic role of IL-6 (69, 71). In support of this possibility, we did not observe any significant increase in the induction of fibrogenic genes in primary HSCs from *Socs1^ΔHSC^* mice following IL-6 stimulation (**Figure 2B**), possibly because SOCS3, which is a more potent regulator of IL-6 signaling than SOCS1 and is necessary to control IL-6 signaling *in vivo* (72), is intact in SOCS1-deficient HSCs.

A direct effect of SOCS1 deficiency in HSCs was the elevated expression of the fibrogenic genes *Acta2*, *Col1a1* and *Timp1*, *Pdgfb* coding for PDGFB and autocrine *Tgfb* induction following TGFβ stimulation (**Figure 2B**), suggesting deregulated TGFβ signaling in SOCS1-deficient HSCs. This idea is supported by increased SMAD3 phosphorylation in the fibrotic livers of *Socs1^ΔHSC^* mice (**Figure 2A**). SMAD3 is considered more crucial for the pro-fibrogenic effect of TGFβ (43, 73, 74). SOCS1 deficiency might potentiate TGFβ signaling in HSCs by multiple mechanisms: In quiescent HSCs, the TGFβ-induced canonical SMAD2/3 pathway is attenuated by the TGFβ pseudoreceptor BAMBI, which is downmodulated by NF-κB that is activated by lipopolysaccharides (LPS) from the gut microbiota via the toll like receptor 4 (TLR4) (75). SOCS1 is a key regulator of TLR4 signaling in macrophages (76, 77). SOCS1 controls LPS-induced NF-κB signaling by virtue of its ability to function as a substrate adaptor for protein ubiquitination. SOCS1 attenuates TLR4 signaling by promoting ubiquitination and proteasomal degradation of a key signaling adaptor the Toll/Interleukin-1 receptor domain containing adaptor protein (TIRAP, also called MAL) (78). Besides, SOCS1 promotes ubiquitination-dependent degradation of p65RelA component of NF-κB itself (79, 80). In addition to the canonical SMAD2/SMAD3 signaling pathway, TGFβ activates JAK1-STAT3 signaling that synergizes with the SMAD3 pathway (81). This pathway can potentially be regulated by SOCS1 via inhibition of JAK1. Clearly, further studies are needed to elucidate the mechanistic basis of SOCS1-dependent regulation of TGFβ signaling in HSCs.

TGFβ stimulation of SOCS1-deficient HSCs increased the induction of *Ccl2*, which is a target gene of NF-κB and encodes MCP-1/CCL2. CCL2 is a key chemoattractant for macrophages, plays a crucial role in liver fibrosis and a potential therapeutic target (12, 82, 83). The fibrotic livers of *Socs1^ΔHSC^* mice also showed elevated expression of *Ccl2* and increased numbers of macrophages (**Figure 3**). Macrophages play a key role in liver homeostasis (45, 84). The normal liver harbors a large reservoir of tissue resident Kupffer cells distributed along the liver sinusoids and monocyte-derived macrophages that are mainly located in the periportal regions. However, hepatic tissue injury causes a large influx of monocyte-derived macrophages that are Ly6C+, express CCR2 and are pro-inflammatory in function. These inflammatory macrophages secrete mediators that attract immune cells, activate HSCs to become myofibroblasts and modulate the ECM, all functions aimed at containing the tissue damage (13). Subsequently, these inflammatory macrophages transition to restorative macrophages that downregulate Ly6C and CCR2 levels, upregulate the chemokine receptor CX3CR1 expression and help clear the ECM and apoptotic HSCs (45). Strikingly, macrophages that express CX3CR1 without downmodulating Ly6C or CCR2 (Ly6C^hi^CCR2+CX3CR1+) accumulate in the fibrotic livers of *Socs1^ΔHSC^* mice (**Figure 5**). These cells likely represent intermediate, transitional state cells that would eventually become pro-restorative Ly6C^lo^CCR2−CX3CR1+ macrophages upon cessation of the fibrotic stimuli. The fact that these cells are discernibly increased in number in the fibrotic livers of control *Socs1^fl/fl^* mice suggests that increased inflammatory response in the livers of *Socs1^ΔHSC^* mice, originating from deregulated cytokine and growth factor signaling in HSCs, hampers this transition. Transcriptomic and proteomic studies on studies on purified HSCs and macrophages from the control and fibrotic livers of *Socs1^fl/fl^ Socs1^ΔHSC^* mice and co-culture experiments using purified HSCs and macrophages would be needed to fully understand how SOCS1-HSCs modulate macrophage phenotype and functions.

Inflammation associated with liver fibrosis is a key driver of HCC development and progression (10, 47, 48). Deletion of Lim homeobox domain 2 (*Lhx2*), a repressor of HSC activation, in HSCs was shown to shift these cells from cytokine-producing phenotype towards the persistently myofibroblastic phenotype and promote HCC development (48). We observed a similarly increased susceptibility of *Socs1^ΔHSC^* mice to liver fibrosis and HCC development. Overall, our findings show that SOCS1 regulates HSC activation by TGFβ and thereby controls liver fibrosis and HCC development at least partly via attenuating pro-inflammatory macrophage recruitment and promoting their transition to restorative macrophages.

## Data availability statement

The original contributions presented in the study are included in the article/**Supplementary Material**. Further inquiries can be directed to the corresponding author.

## Ethics statement

The animal study was approved by Université de Sherbrooke Ethics Committee for Animal Care and Use. The study was conducted in accordance with the local legislation and institutional requirements.

## Author contributions

RK: Formal Analysis, Investigation, Methodology, Writing – original draft. MY: Investigation, Methodology, Writing – review & editing. AY: Writing – review & editing, Resources. AM: Writing – review & editing, Funding acquisition, Investigation, Methodology, Supervision. SR: Investigation, Methodology, Writing – review & editing, Conceptualization, Formal Analysis, Resources, Validation. SI: Conceptualization, Formal Analysis, Investigation, Methodology, Resources, Validation, Writing – review & editing, Data curation, Funding acquisition, Project administration, Supervision, Visualization, Writing – original draft.
