# Supplementary material for: Hepatic stellate cell-intrinsic role of SOCS1 in controlling hepatic fibrogenic response and the pro-inflammatory macrophage compartment during liver fibrosis
Source: Front Immunol. 2023 Oct 4;14:1259246. doi: 10.3389/fimmu.2023.1259246 (PMC10582746; doi:10.3389/fimmu.2023.1259246)
Supplement: Supplementary file 1 [file Table_1.pdf]

Hepatic stellate cell-intrinsic role of SOCS1 in controlling hepatic fibrogenic response and the pro-inflammatory macrophage compartment during liver fibrosis

Kandhi et al.,

Supplementary Table S1. Antibodies used for immunohistochemistry or immunofluorescence.

Antibody	Supplier	Clone	Cat. #
αSMA	Cell Signaling technology	D4K9N (Rabbit mAb)	19245S
CD68	Abcam	Rabbit polyclonal Ab	125212
Desmin	Abcam	Rabbit polyclonal Ab	Ab15200

Supplementary Table S2. List of RT-qPCR primers.

Gene name	Gene ID	Sense primer	Anti-sense primer	Amplicon Size (bp)
Acta2	NM_007392.3	AGTAATGGTTGGAATGG	GTGTCGGATGCTCTTCAGG	146
Ccl2	NM_011333.3	CAGGTCCCTGTCATGCTTCT	GTGGGGCATTAACTGCAT	144
Ccl5	NM_013653.3	TGCAGAGGACTCTGAGACAGC	GAGTGGTGTCCGAGCCATA	129
Col1a1	NM_007742.4	CTCCCAGAACATCACCTATCAC	ACTGTCTTGCCCCAAGTTCCG	150
Col3a1	NM_009930.2	AAGTCAAGGAGAAAGTGGTCG	CAGTCTCCCCATTCTTTCCAG	149
Cx3cl1	NM_009142.3	TCTTCCATTTGTGTACTCTGCT	GGACTCCTGGTTTAGCTGATAG	128
Il1b	NM_008361.4	TCCTGTGTAATGAAAGACGGC	ACTCCACTTTGCTCTTGACTTC	139
Il6	NM_031168.2	AGTCCGGAGAGGAGACTTCA	TTGCCATTGCACAACTCTTT	150
Mmp2	NM_008610.3	CAAGTTCCCCGGCGATGTC	TTCTGGTCAAGGTCACCTGTC	91
Mmp9	NM_013599.3	GATCCCCAGAGCGTCATTC	CCACCTTGTTCACCTCATTTTG	129
Pdgfb	NM_011057.4	CCTGCAAGTGTGAGACAGTAG	CTTTCGGTGCTTGCCTTTG	146
Timp1	NM_011593.2	TTGCATCTCTGGCATCTGG	TGGTCTCGTTGATTTCTGGG	136
Tgfb1	NM_011577.2	ATACGCCTGAGTGGCTGTCT	CTGATCCCGTTGATTTCCA	124
36B4 (Rplp0)	NM_007475.5	TCTGGAGGGTGTCCGCAA	CTTGACCTTTTCAGTAAGTGG	148

Supplementary Table S3: List of antibodies used for western blot.

Name	Supplier	Cat no.	Clone no.
α-SMA	Cell Signaling Technology	19245S	D4K9N (Rabbit mAb)
β-actin	Cell Signaling Technology	4970S	13E5
Collagen 1	Abcam	15200	Rabbit polyclonal Ab
Phospho-ERK1/2	Cell Signaling Technology	4377	197G2 (Rabbit mAb)
ERK1/2	Cell Signaling Technology	9102	Rabbit polyclonal Ab
Phospho-SMAD2 (Ser465/467)	Cell Signaling Technology	3108L	138D4
SMAD2	Cell Signaling Technology	3103S	L16D3
Phospho-SMAD3 (Ser423/425)	Cell Signaling Technology	9520S	C25A9
SMAD3	Cell Signaling Technology	9513S	Rabbit polyclonal Ab

Supplementary Table S4. Antibodies used for Flow cytometry:

A) Myeloid cell panel

**Antibody**	**Fluorochrome**	**Source**	**Clone**	**#Cat.**
Fixable Viability Dye	e-fluor 780	ebiosciences		65-0865
CD45	Brilliant Violet 510	Biolegend	30-F11	103138
CD11b	eFluor450	ebiosciences	M1/70	100353
Ly6G	PerCP	Biolegend	1A8	127654
Ly6C	FITC	eBiosciences	HK1.4	53-5932-82
CD11c	Alexa Fluor 700	ebiosciences	N418	5016869
CCR2	PE	R&D Systems	475301 (Rat mAb)	FAB5538P
CX3CR1	APC	Biolegend	SA011F11	149008

B) Lymphoid cell panel

**Antibody**	**Fluorochrome**	**Source**	**Clone**	**#Cat.**
CD45	Brilliant Violet 605	Biolegend	30-F11	103140
CD3ε	Brilliant Violet 510	Biolegend	145-2C11	100353
TCRβ	PE/Dazzle594	Biolegend	109240	H57-597
CD4	Alexa Fluor 700	ebiosciences	GK1.5	5016851
CD8α	eFluor450	ebiosciences	53-6.7	48-0081-82
CD62L	APC	ebiosciences	MEL-14	17-0621-83
CD44	FITC	Biolegend	IM7	103006
CD69	PE-Cy7	Biolegend	H1.2F3	104512
NK1.1	APC-Cy7	Biolegend	PK136	108724
