# Supplementary material for: Hepatic stellate cell-intrinsic role of SOCS1 in controlling hepatic fibrogenic response and the pro-inflammatory macrophage compartment during liver fibrosis
Source: Front Immunol. 2023 Oct 4;14:1259246. doi: 10.3389/fimmu.2023.1259246 (PMC10582746; doi:10.3389/fimmu.2023.1259246)
Supplement: Supplementary file 2 [file DataSheet_1.pdf]

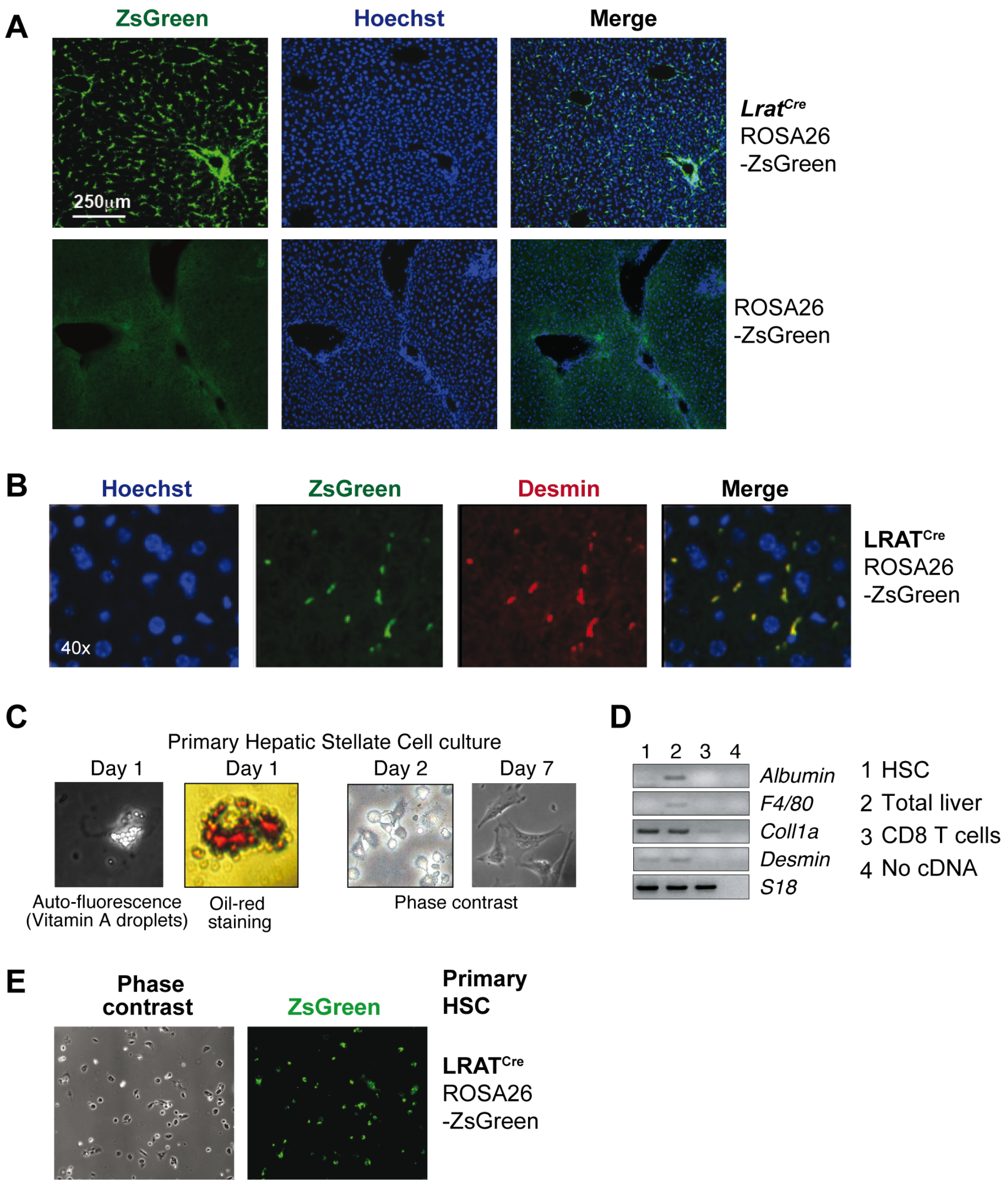

Supplementary Figure S1. *Lrat* promoter driven Cre expression in HSCs.
(A) Fluorescence imaging of liver sections showing distribution of ZsGreen positive HSCs in the liver parenchyma of *Lrat*^{Cre}-ROSA26-ZsGreen mice but not in control ROSA26-ZsGreen mice.
(B) Co-localisation of Zs-Green fluorescence with desmin-positive cells in the liver sections of *Lrat*^{Cre} ROSA26-ZsGreen mice.
(C) Primary HSC cultures from wildtype C57BL/6 mice showing autofluorescence and Oil-Red staining on day 1 after culture initiation, and the loss of autofluorescence and the gain of fibroblast morphology after prolonged culture.
(D) HSC enrichment shown by the expression of *Col1a1* and *Desmin* genes but not that of hepatocyte- (*Albumin*) and macrophage- (F4/80) specific genes.
(E) Expression of ZsGreen in HSCs from *Lrat*^{Cre} ROSA26-ZsGreen mice one day after cell isolation and culture.

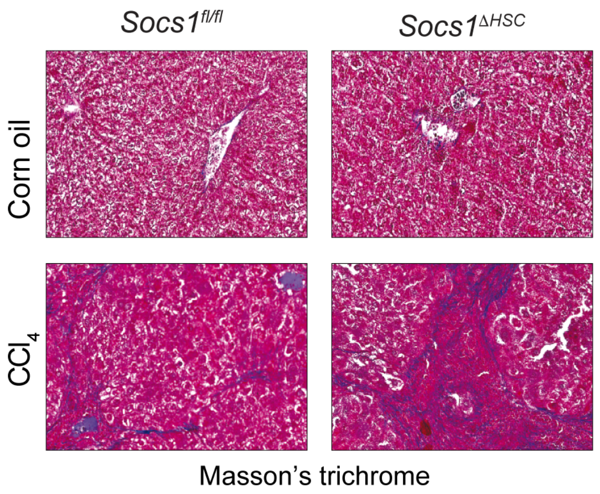

Supplementary Figure S2. SOCS1 deficiency in HSCs potentiates CCl_4-induced liver fibrosis. Masson's trichrome staining of representative liver sections from *Socs1*$^{\Delta HSC}$ and *Socs1*$^{fl/fl}$ treated with CCl_4 or vehicle (corn oil).

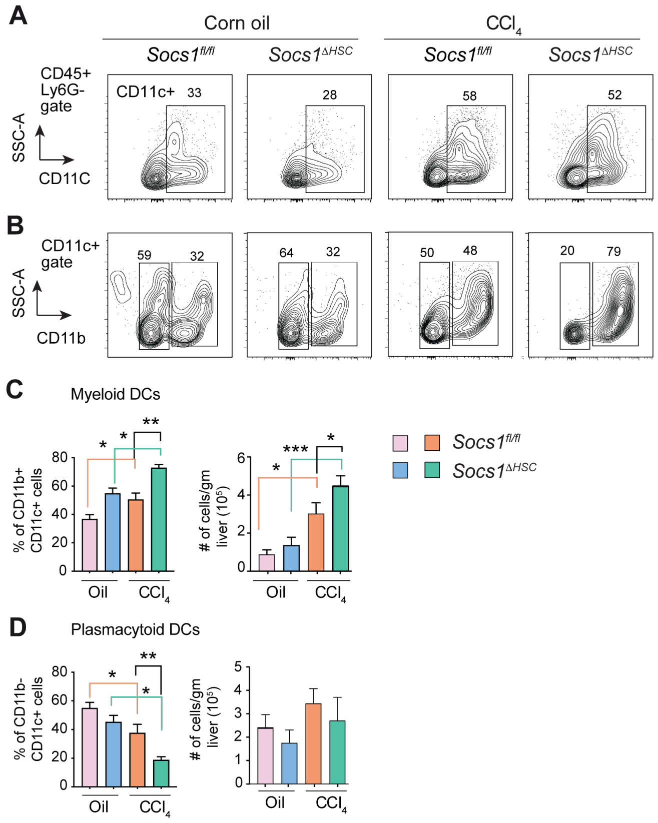

Supplementary Figure S3. Fibrotic livers of HSC-specific SOCS1 deficient mice harbor increased numbers of myeloid-derived dendritic cells. (A,B) Representative zebra blots showing the expression of (A) CD11c within the CD45+ Ly6G− cells and (B) CD11b within CD11c+ cells in the livers of *Socs1*$^{\Delta HSC}$ and *Socs1*$^{fl/fl}$ control mice. Numbers inside the plots indicate the cell proportions within the indicated gates. (C) Proportions and absolute counts of CD11b+CD11c+ myeloid derived dendritic cells (DCs). (D) Proportions and absolute counts of CD11b−CD11c+ plasmacytoid DCs. Pooled data from 4-6 mice per group from two different experiments are shown (mean ± SE). One-way ANOVA with Tukey's multiple comparison test. * *p* <0.05, ** *p* <0.01, *** *p* <0.0001.

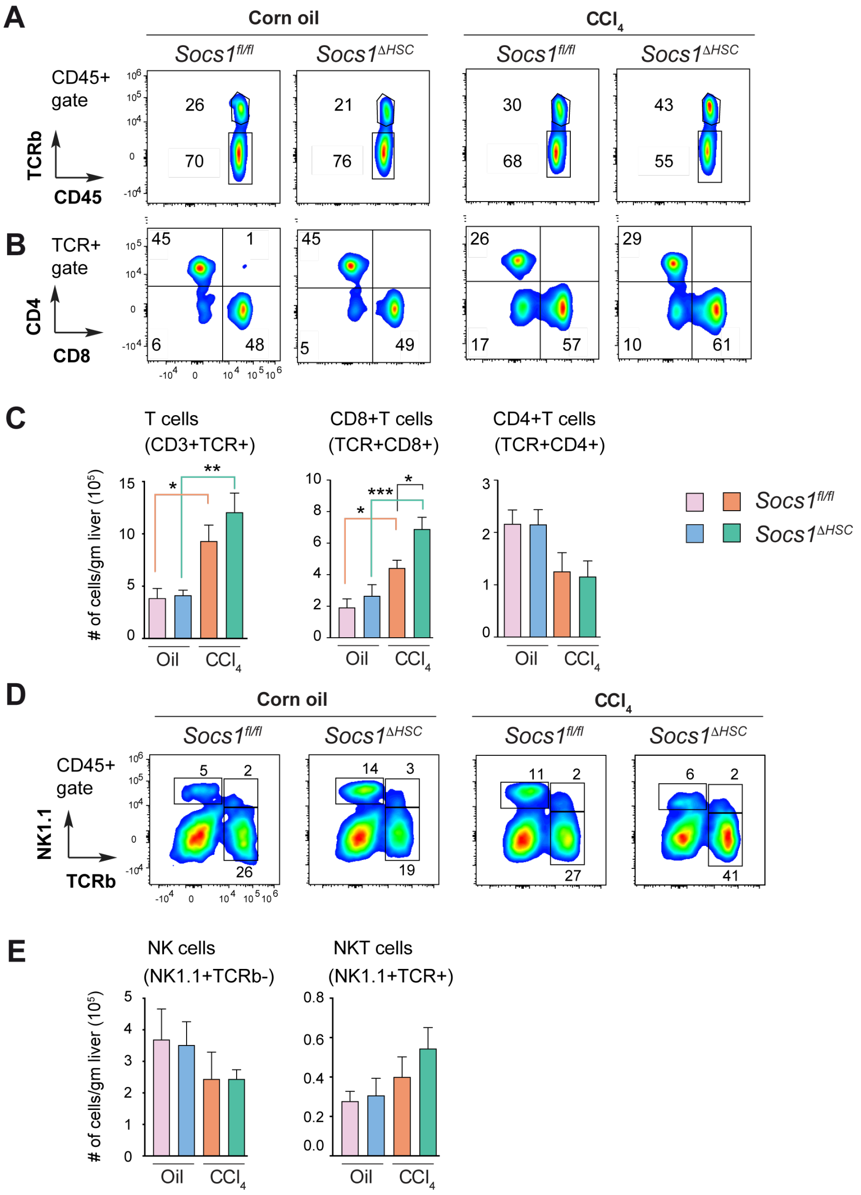

Supplementary Figure S4. Fibrotic livers of HSC-specific SOCS1 deficient mice accumulate CD8+ T lymphocytes. (A,B) Representative density blots showing the expression of (A) TCRb within the CD45+ cells and (B) CD4 and CD8 within TCRb+ cells in the livers of *Socs1*$^{\Delta HSC}$ and *Socs1*$^{fl/fl}$ control mice. Numbers inside the plots indicate the cell proportions within the indicated gates or quadrants. (C) Absolute counts of total TCR+, CD8+ and CD4+ T cells. (D) NK and NKT cell profiles within IHLs from representative mice of the indicated groups. (E) Absolute counts. Data was pooled from 4-6 mice per group (mean ± SE). One-way ANOVA with Tukey's multiple comparison test. * p <0.05, ** p <0.01, *** p <0.0001.

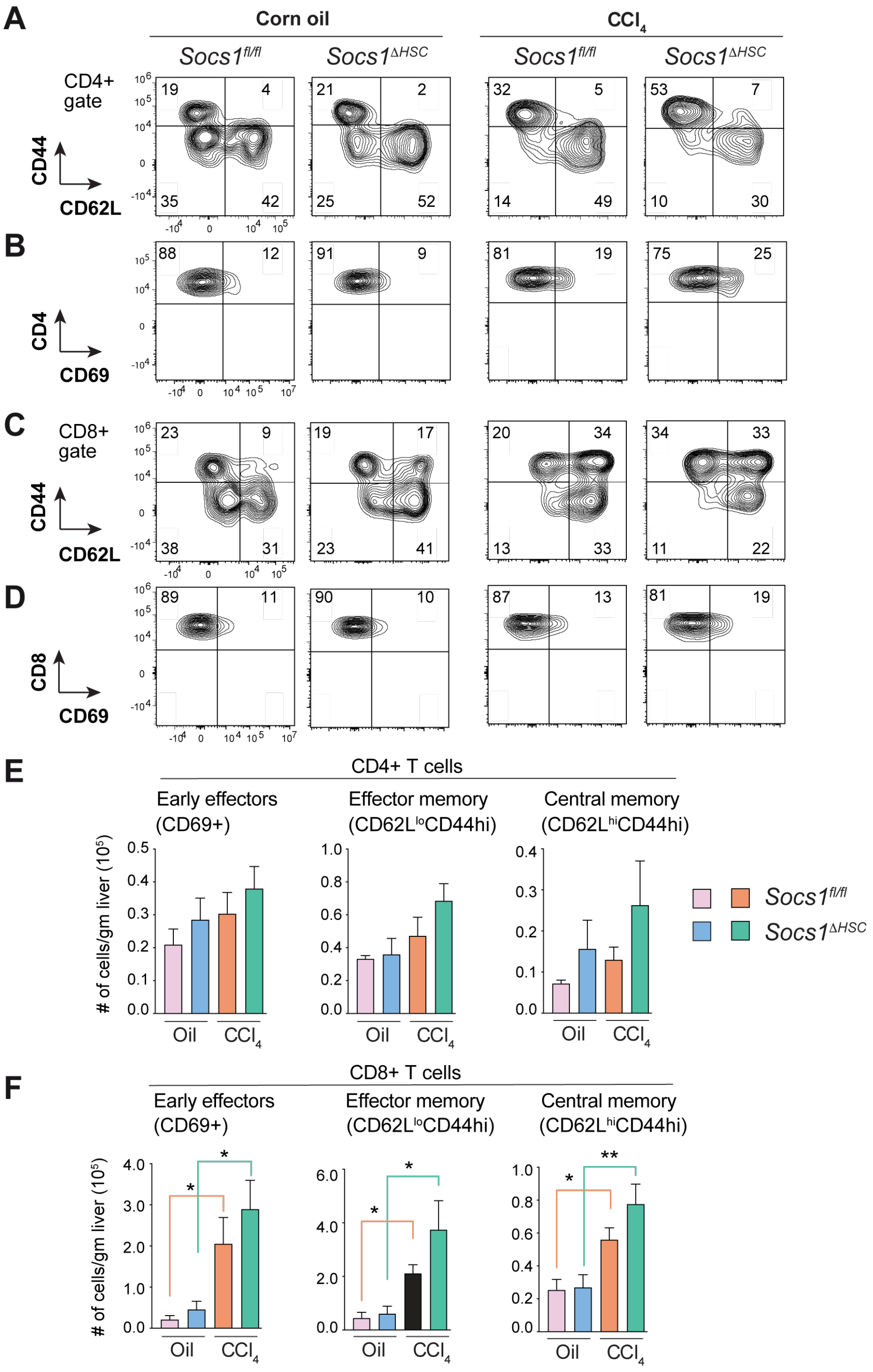

Supplementary Figure S5. CD8+ T cells in the fibrotic livers of HSC-specific SOCS1 deficient mice show activated phenotype. (A-D) Representative density blots showing the expression of (A) CD44, CD62L (A,C) and CD69 (B,D) within CD4+ (A,B) and CD8+ (C,D) T lymphocytes in the livers of *Socs1*$^{\Delta HSC}$ and *Socs1*$^{fl/fl}$ control mice. Numbers inside the plots indicate the cell proportions within the quadrants. (E,F) Absolute counts of CD69+ effector, CD44^{hi}CD62L^{lo} effector memory and CD44^{hi}CD62L^{hi} central memory cells within CD4+ (F) and CD8+ (G) T lymphocytes. Data was pooled from 4-6 mice per group (mean ± SE). One-way ANOVA with Tukey's multiple comparison test. * p <0.05, ** p <0.01.
